# Optimisation of acid hydrolysis conditions of choline esters and mass spectrometric determination of total choline in various foods

**DOI:** 10.1038/s41598-024-69008-z

**Published:** 2024-08-02

**Authors:** Yoshinari Hirakawa, Kazuhiro Fujita, Masako Katayama, Toshiaki Yokozeki, Yushi Takahashi, Izumi Yoshida, Kiyotaka Nakagawa

**Affiliations:** 1 Japan Food Research Laboratories, 7-4-41 Saitoasagi, Ibaraki, Osaka 567-0085 Japan; 2 Laboratory of Food Function Analysis, Graduate School of Agricultural Science, Tohoku University, 468-1 Aramaki Aza Aoba, Aoba-Ku, Sendai, 980–8572 Japan

**Keywords:** Choline, Total choline, Choline esters, Acid hydrolysis, Liquid chromatograph-tandem mass spectrometry, Various foods, Nutrition, Small molecules

## Abstract

Determining the content of the nutrient choline in foods and obtaining the required amount from the diet are crucial. One way to measure choline in foods is by converting choline esters to free choline via acid hydrolysis, followed by quantifying the total choline, as adopted by the AOAC method (*AOAC-Choline*); however, certain choline esters are difficult to hydrolyse. Here, we investigated various acid hydrolysis conditions to establish a reliable method for determining the total choline in foods by detecting free choline using highly sensitive and selective mass spectrometry. Hydrolysis in 0.055 mol/L HCl for 8 h in an autoclave (121 °C) was found to be optimal for the hydrolysis of choline esters in various foods. Twenty-four foods, including grains, seed, vegetables, fruits, mushroom, algae, fish, meats, beverage, processed foods, and egg, were measured. The trends in the total choline content were consistent with previous reports; however, the choline content was 10–20% higher than that measured using *AOAC-Choline*. Therefore, re-evaluation of the total choline content in foods using our constructed method is recommended. This reassessment will allow for a more reliable determination of choline intake for maintaining health.

## Introduction

**Figure 1:**
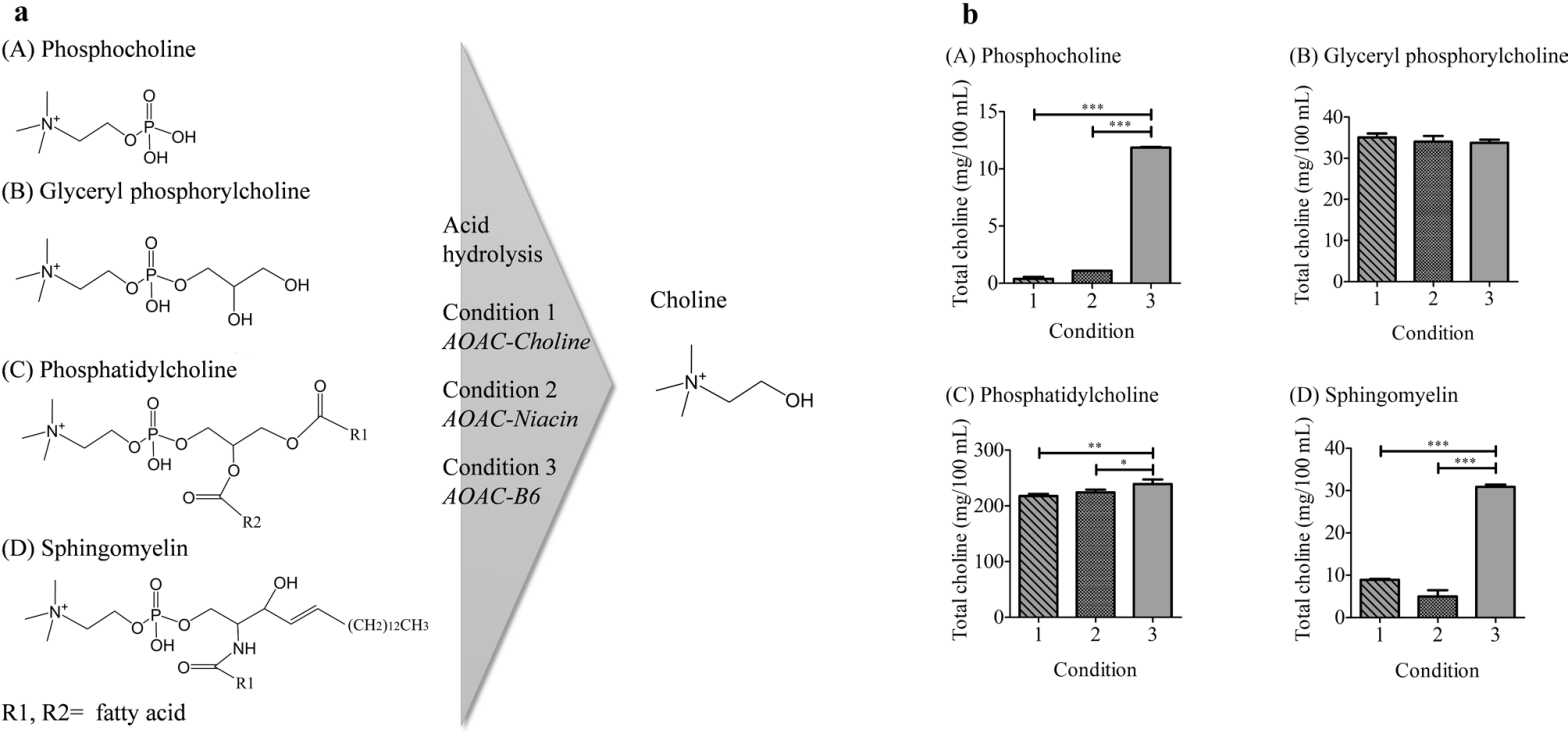
(**a**) Structure of choline esters and their acid hydrolysis under three conditions. (**b**) Preliminary studies on conditions for hydrolysis of choline esters. Each test solution ((**A**) 50.00 mg/100 mL phosphocholine, (**B**) 90.60 mg/100 mL glycerophosphorylcholine, (**C**) 1985 mg/100 mL phosphatidylcholine, or (**D**) 356.2 mg/100 mL sphingomyelin) was hydrolysed under three different conditions. After hydrolysis, the solution was analysed by LC–MS/MS. Detailed conditions are described in the Materials and methods. The data represent the mean ± SD (*n* = 3). Significant differences from Condition 3 were analysed using one-way ANOVA followed by Dunnett's test (^*^P < 0.05, ^**^P < 0.01, ^***^P < 0.001).

Choline is an important water-soluble substance with various functions in the human body. In vivo, choline functions primarily in the form of esters such as phosphocholine, glyceryl phosphorylcholine, phosphatidylcholine, and sphingomyelin (Fig. 1a), which are essential, especially for maintaining the cell membrane and neurotransmission^1,2^. Despite its importance, the amount of choline synthesised in the human body is low, and it is rarely synthesised in organs other than the liver. Therefore, choline must be obtained through diet. Foods high in choline include raw chicken liver and egg yolk. The current recommended daily choline intake is 425 mg/day for adult women and 550 mg/day for adult men and lactating women^3,4^. Reports indicate that insufficient dietary choline intake leads to various health issues, such as liver dysfunction, renal failure, abnormal bone formation, and growth disorders^2^. Therefore, determining the total choline content of foods and obtaining the required amount are important for maintaining health.

Two primary analytical approaches have been reported for determining the amount of total choline in foods. One method is quantifying individual choline esters and adding them to obtain the total choline content^5,6^. The total choline content of foods calculated using this method are listed in the United States Department of Agriculture (USDA) database; herein, this method is referred to as *USDA-Choline*. Information on the individual and total choline content determined by *USDA-Choline* is useful for understanding the metabolism of individual choline by the body and determining the most bioavailable forms. However, *USDA-Choline* requires a separate analysis of each choline, making it labour-intensive. Another method uses acids, such as hydrochloric acid (HCl) and nitric acid (HNO_3_), to convert choline esters into free choline during the extraction process and quantifies choline as total choline^7,8^. This method was adopted by the AOAC official methods for choline and is referred to herein as *AOAC-Choline*. The conversion of choline esters into free choline takes some time, but as an advantage, only free choline needs to be measured once the hydrolysis is completed. However, it has been suggested that the hydrolysis of acid-stable phosphocholine remains insufficient^9,10^. Perhaps because of this concern, *AOAC-Choline* has been applied only to certain foods (e.g., infant formula and related foods).

This study aims to address the possible insufficient hydrolysis of choline and determine the optimal conditions for hydrolysing choline esters in various foods by referring to acid hydrolysis conditions in *AOAC-Choline*^8^ and even other vitamins’ AOAC methods (*AOAC-Niacin*^11^ and *AOAC-B6*^12^) (Supplementary Fig. S1 online). In addition, using liquid chromatography-tandem mass spectrometry (LC–MS/MS) or liquid chromatography-mass spectrometry (LC–MS), which can measure choline (free form) with high sensitivity and selectivity, our ultimate goal is to establish a highly accurate method for measuring the total choline in various foods under conditions of adequate choline ester hydrolysis.

## Materials and methods

### Reagents and food samples

Choline chloride (100%) was purchased from the United States Pharmacopoeia (MD, USA). Choline-1,1,2,2-d4 chloride (99.1%) was obtained from CDN ISOTOPES (Point Claire, Canada). Phosphocholine chloride calcium salt tetrahydrate (≥ 98%), soybean glyceryl phosphorylcholine (≥ 98%, L-α-glycerylphosphorylcholine), egg yolk phosphatidylcholine (≥ 98%), and egg yolk sphingomyelin (≥ 98%) were purchased from Toronto Research Chemicals, Inc. (North York, Canada), Sigma-Aldrich (MO, USA), Nacalai Tesque, Inc. (Kyoto, Japan), and Nagara Science Co., Ltd. (Gifu, Japan), respectively. Milli-Q ultrapure water (18.2 MΩ cm) was used. All the other reagents were of analytical grade.

Grains (wheat flour and soybean powder), seed (pistachio nuts), vegetables (raw tomato, raw carrot, raw spinach, and raw eggplant), fruits (raw apple and 100% orange juice), mushroom (dried shiitake mushroom), algae (dried seaweed), fish (raw fish salmon and tarako (salted cod roe)), meats (raw pork fresh loin and raw chicken liver), beverage (bottled green tea), processed foods (honey, adult nutritional ready-to-feed, infant liquid milk, infant formula, yogurt and fruit granola), and egg (egg yolk) were purchased from a local supermarket and pharmacy in Osaka, Japan, in the summer of 2022. Certified reference material for infant and adult nutritional formulas (NIST 1869) was obtained from the National Institute of Standards and Technology (MD, USA). Each liquid (100% orange juice, bottled green tea, honey, and infant liquid milk) was mixed thoroughly to ensure homogeneity, whereas the solid foods were individually and finely ground using a grinder (Force Mill, Osaka Chemical Co., Ltd., Osaka, Japan). The food samples were stored in a freezer (MPR-715F, PHC Co., Ltd., Tokyo, Japan) at −30 °C until analysis.

### Preliminary studies on hydrolysis conditions

Phosphocholine and glyceryl phosphorylcholine were individually dissolved in water at concentrations of 50.00 and 90.60 mg/100 mL, respectively. Phosphatidylcholine and sphingomyelin were each prepared in ethanol at concentrations of 1985 and 356.2 mg/100 mL, respectively. Using each solution as a test solution, preliminary hydrolysis studies were conducted under three conditions. Condition 1 (based on *AOAC-Choline*^8^): A 1 mL aliquot of the test solution was collected in a polytetrafluoroethylene vessel (100 mL) and mixed with 25 mL of 3.650 mol/L HNO_3_ aqueous solution. Hydrolysis was performed in a microwave (ETHOS One, Milestone-MLS, Leutkirch, Germany) at 1000 W. The temperature was increased to 120 °C in 10 min and maintained for 40 min. Condition 2 (based on *AOAC-Niacin*^11^): A 1 mL aliquot of the test solution was placed in an Erlenmeyer flask (100 mL) and mixed with 50 mL of 0.500 mol/L sulfuric acid (H_2_SO_4_) aqueous solution. The hydrolysis was carried out using an autoclave (LSX-500, Tomy Digital Biology Co., Ltd., Tokyo, Japan) at 121 °C for 30 min. Condition 3 (based on *AOAC-B6*^12^): A 1 mL aliquot of the test solution was placed in an Erlenmeyer flask (300 mL) and mixed with 70 mL of 0.055 mol/L HCl aqueous solution. The hydrolysis was conducted in an autoclave at 121 °C for 4 h.

After cooling the hydrolysate from Condition 1, approximately 11 mL of 10.00 mol/L sodium hydroxide (NaOH) aqueous solution was added, followed by the dropwise addition of 1.000 mol/L NaOH until reaching a pH of 6.8 for neutralising the solution containing a high acid concentration. The resulting solution was made up to 100 mL with water and filtered through a filter paper (No. 2, Toyo Roshi Kaisha, Ltd., Tokyo, Japan). A 1 mL aliquot of the diluted solution and 0.25 mL of choline-1,1,2,2-d4 chloride solution (1.000 μg/mL water) as an internal standard (IS) were transferred to a flask, and water was added to achieve a volume of 5 mL. A 4 mL aliquot of the solution (IS concentration: 0.050 μg/mL; assumed choline concentration: 0.025‒0.150 μg/mL) was loaded onto a solid-phase column (InertSep WCX-FF, 60 mg/3 mL, GL Sciences, Tokyo, Japan) preconditioned with 5 mL of 0.010 mol/L phosphate-buffered saline (pH 6.8). After washing with 4 mL of water, free-form choline was eluted with 4 mL of a 0.1% formic acid aqueous solution and adjusted to a final volume of 5 mL with 0.1% formic acid. For the hydrolysate from Condition 2, after cooling, approximately 5 mL of 10.00 mol/L NaOH was added, followed by the dropwise addition of 1.000 mol/L NaOH to adjust the pH to 6.8. Solid-phase extraction was performed similar to Condition 1. For the hydrolysate from Condition 3, after cooling, the hydrolysate was made up to 100 mL with water. Because the pH was not so low, the hydrolysate was filtered through a filter paper without neutralisation (solid-phase extraction was not performed because there was no effect of the neutralised salt). A portion of the solution was diluted 5–1000-fold using 0.1% formic acid. A 0.9 mL solution was mixed with a 0.1 mL IS solution to achieve a 1 mL final solution (IS concentration: 0.040 μg/mL; assumed choline concentration: 0.020‒0.120 μg/mL).

### Optimising hydrolysis conditions

Focusing on Condition 3, the optimum hydrolysis conditions were investigated by varying the HCl concentration (0.010, 0.055, and 0.100 mol/L) and hydrolysis time (0.5, 4, 8, and 16 h). Once the optimal HCl concentration was determined, hydrolysis tests were conducted using foods rich in choline esters. Briefly, a crushed food sample (egg yolk, raw chicken liver, soybean powder, or pistachio nuts; 0.5 g each) was placed in an Erlenmeyer flask (300 mL) and hydrolysed for different times (4, 8, and 16 h) with the optimum HCl concentration.

### Analytical conditions for LC–MS/MS and validation of quantitative performance

The hydrolysed sample (1 mL) was filtered through a 0.45 μm polytetrafluoroethylene membrane filter (Toyo Roshi Kaisha, Ltd., Tokyo, Japan). The filtrate (1 μL) was injected into the LC–MS/MS instrument to determine the free choline. The LC–MS/MS instrument comprised a 1260 Infinity II Prime LC System (Agilent Technologies, Palo Alto, CA, USA) and an Ultivo Triple Quadrupole detector. To realize choline detection with high sensitivity and selectivity, LC–MS/MS measurements were performed by referring to previous method^13^. An InertSustain pentafluorophenyl (PFP) column (2.0 × 150 mm i.d., 3 μm; GL Sciences, Tokyo, Japan) was used with a mixture of acetonitrile and water (50:50, v/v; containing 0.1% formic acid) as the mobile phase at a flow rate of 0.2 mL/min. The column temperature was 40 °C. MS/MS analysis was conducted in electrospray ionisation positive-ion mode (gas flow and temperature: 10 L/min and 300 °C, respectively; nebuliser pressure: 50 psi; sheath gas flow and temperature: 11 L/min and 250 °C, respectively; capillary voltage: 3500 V; nozzle voltage: 1000 V). The optimal fragmentation voltage was 80 V. Free choline was detected in the selected reaction monitoring (SRM) mode: *m/z* 104.2 → 60.1. For IS, *m/z* 108.2 → 60.0 was used. The collision energy was 17 eV for all SRM.

To evaluate the validity of the quantitative values of choline in various foods, 24 crushed and mixed samples were placed in Erlenmeyer flasks (300 mL). The amount of food used for the measurement was 0.5 g. For raw tomato, raw carrot, raw spinach, raw eggplant, raw apple, 100% orange juice, bottled green tea, and honey, 1.0 g each was used. After visual confirmation of the homogeneity of the samples, they were hydrolysed under optimal conditions and subjected to LC–MS/MS analysis. The choline content of the food sample was calculated (Supplementary Fig. S1 online) using a calibration curve prepared as follows: Choline chloride was dried at 102 °C for 2 h, accurately weighing it, and dissolving it in water to prepare a stock solution of 2000 μg/mL. The solution was diluted with 0.1% formic acid to achieve concentrations of 0.005, 0.010, 0.020, 0.040, 0.080, 0.120, and 0.160 μg/mL, to which IS was added at a concentration of 0.040 μg/mL and analysed by LC–MS/MS.

According to the AOAC guidelines for the single-laboratory validation of chemical methods for dietary supplements and botanicals^14^, the linearity of the calibration curve was examined using the coefficient of determination (Supplementary Table S1 online). Precision (*n* = 10) was determined by performing the analyses twice per day for five days to assess laboratory reproducibility (RSD_wr_) and HorRat_r_ values. Accuracy (*n* = 3) was evaluated by spiking the samples used in the precision study at a single level (100% of the choline level of the unspiked sample) and analysing them in triplicate. The limit of quantitation (LOQ) and limit of detection (LOD) were calculated from the standard deviation (SD) of low-concentration sample (bottled green tea). LOD and LOQ were determined as 3 × and 10 × SD, respectively.

### Analytical conditions for LC–MS

LC–MS analysis was performed using an LCMS-2050 coupled with a Nexera LC system (Shimadzu, Kyoto, Japan). The LC conditions were the same as those used for LC–MS/MS. MS analysis was conducted in dual-ion-source positive-ion mode (desolvation temperature: 450 °C; desolvation line temperature: 250 °C; interface voltage: 3.5 V; detection voltage: 0.95 V). Based on a previous study^9^, free choline was detected in selected ion monitoring (SIM) mode at *m/z* 104.2. For the IS, *m/z* 108.2 was used.

### Statistical analysis

For the hydrolysis studies, each analysis was performed in triplicate. The data are expressed as mean ± SD (*n* = 3) and analysed by one-way analysis of variance (ANOVA), followed by Dunnett’s test using GraphPad Prism (GraphPad Software, San Diego, CA, USA). For the validation of quantification performance (i.e., total choline determination of 24 food samples), each analysis was performed 10 times, and the data are expressed as mean ± SD (*n* = 10). For total choline measurement of food samples using *AOAC-Choline*, each analysis was performed 3 times, and the data are expressed as mean ± SD (*n* = 3). These choline data in food samples were assessed by an unpaired *t*-test with Welch’s correction. *P* < 0.05 was considered statistically significant.

## Results and discussion

### Preliminary studies on hydrolysis conditions for choline esters

Previous studies have suggested that the conditions for hydrolysing choline esters (e.g., acid concentration, hydrolysis temperature, and equipment used) affect the extent of hydrolysis^15^. Thus, the acid hydrolysis conditions for the effective conversion of acid-stable phosphocholine and other choline esters into free choline must be established to accurately determine the total choline content in various food samples. In this study, a test solution containing either phosphocholine, glyceryl phosphorylcholine, phosphatidylcholine, or sphingomyelin was hydrolysed under three different conditions (Supplementary Fig. S1 online) based on the acid hydrolysis conditions in *AOAC-Choline*^8^ and even other vitamins’ methods (*AOAC-Niacin*^11^ and *AOAC-B6*^12^). The choline ester concentrations in the test solutions were set to match those expected from actual food samples. After hydrolysis, the free choline content was measured using sensitive and selective LC–MS/MS to determine the hydrolysis rate (see below for the LC–MS/MS analytical conditions). The hydrolysis rate differed depending on the reaction conditions (Fig. 1b). Unexpectedly, under Condition 3, which has the lowest acid concentration of 0.055 mol/L HCl, the hydrolysis rate of choline esters, particularly phosphocholine and sphingomyelin, was higher than that of other conditions.

### Optimisation of hydrolysis conditions for choline esters

**Figure 2:**
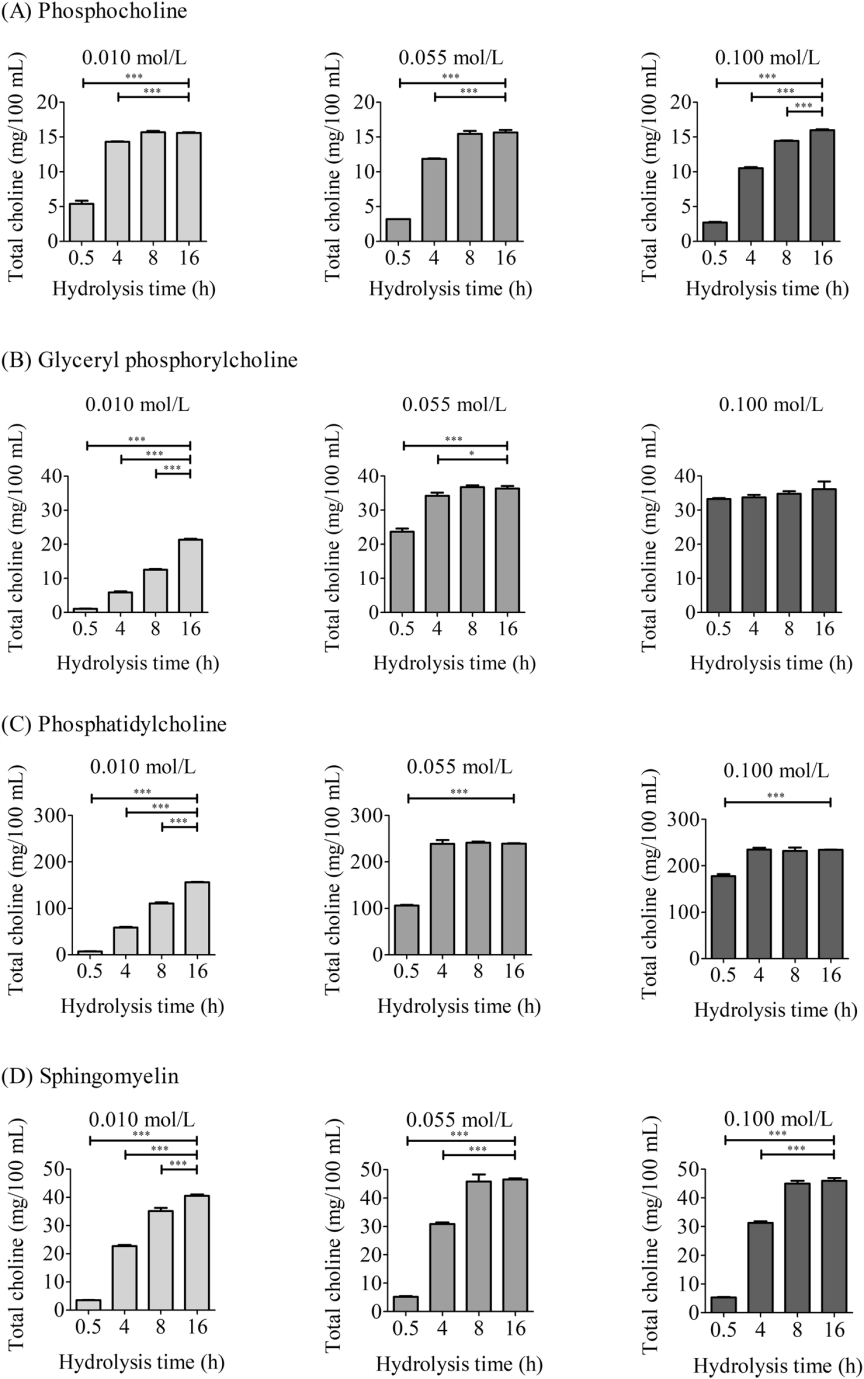
Optimisation of hydrolysis conditions using test solutions. Each test solution ((**A**) 50.00 mg/100 mL phosphocholine, (**B**) 90.60 mg/100 mL glyceryl phosphorylcholine, (**C**) 1985 mg/100 mL phosphatidylcholine, or (**D**) 356.2 mg/100 mL sphingomyelin) was hydrolysed under Condition 3 with varying HCl concentrations and hydrolysis times. After hydrolysis, the solution was analysed by LC–MS/MS. Detailed conditions are described in the Materials and methods. The data represent the mean ± SD (*n* = 3). Significant differences from 16 h were analysed using one-way ANOVA followed by Dunnett's test (^*^P < 0.05, ^***^P < 0.001).

**Figure 3:**
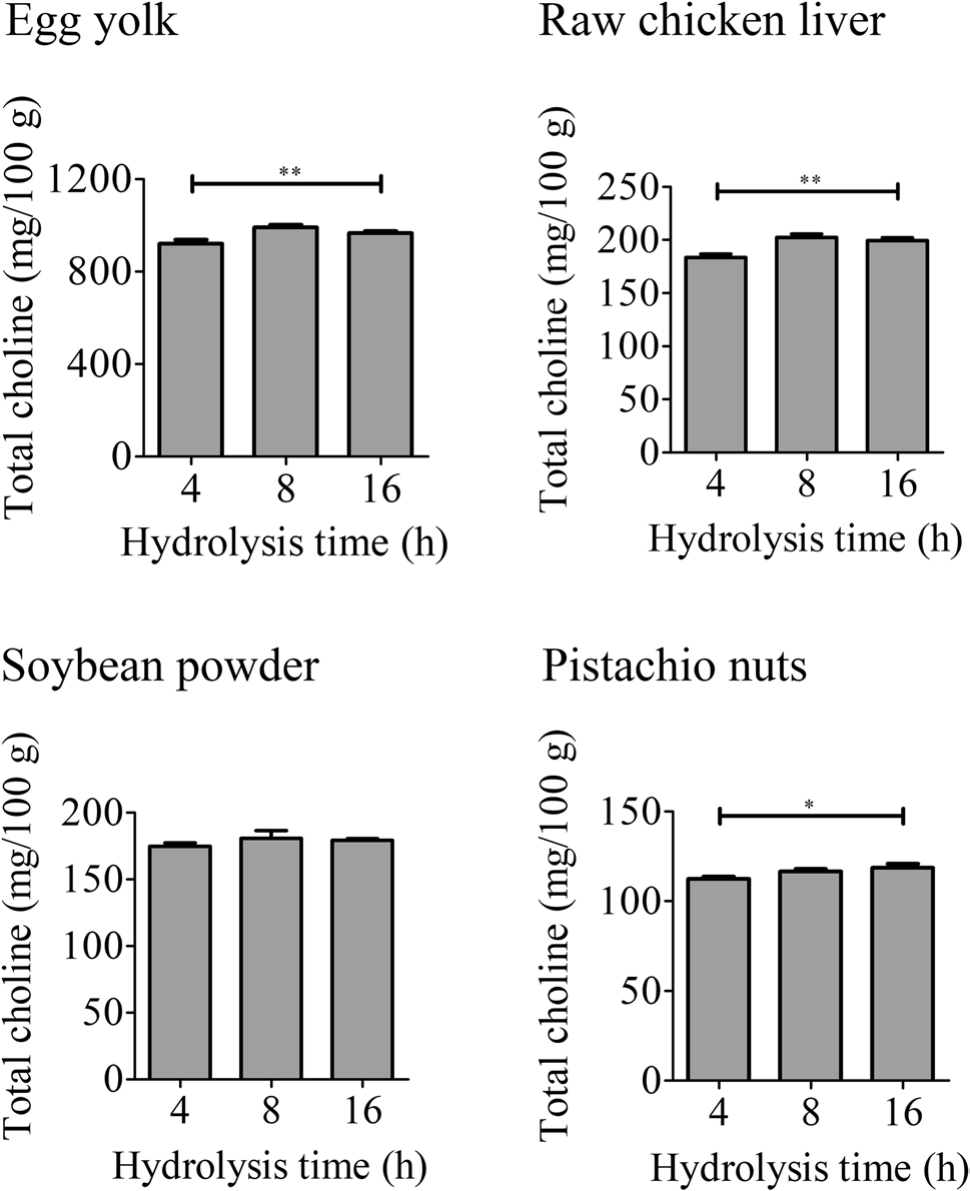
Optimisation of hydrolysis conditions using food samples. Each food sample (egg yolk, raw chicken liver, soybean powder, and pistachio nuts) was hydrolysed under Condition 3 with varying hydrolysis times. After hydrolysis, the solution was analysed by LC–MS/MS. Detailed conditions are described in the Materials and methods. The data represent the mean ± SD (*n* = 3). Significant differences from 16 h were analysed using one-way ANOVA followed by Dunnett's test (^*^P < 0.05, ^**^P < 0.01).

Using the above low acid concentration (0.055 mol/L HCl) in Condition 3 as the baseline, different acid concentrations (0.010, 0.055, and 0.100 mol/L) were tested to further optimise the acid concentration (Supplementary Fig. S1 online). Different treatment times (0.5, 4, 8, and 16 h) were also evaluated. Supporting the results of the above preliminary study, the hydrolysis of phosphocholine proceeded at low acid concentration (0.055 mol/L) and even at a lower concentration (0.010 mol/L). Phosphocholine was completely hydrolysed after autoclaving at 121 °C for 8 h at these acid concentrations (Fig. 2). Similarly, glyceryl phosphorylcholine, phosphatidylcholine, and sphingomyelin were hydrolysed for 8 h at a low acid concentration (0.055 mol/L). Based on these results, hydrolysis tests were conducted using foods rich in major choline esters (egg yolk, raw chicken liver, soybean powder, and pistachio nuts). Comparing the results obtained by treatment for 4, 8, and 16 h, hydrolysis for 4 h was deemed inadequate; therefore, hydrolysis in 0.055 mol/L HCl for 8 h in an autoclave (121 °C) was optimal (Fig. 3).

Just like the present study, acid treatment at a low concentration (0.055 mol/L) is also an effective method for hydrolysing pyridoxal monophosphate, the phosphate form of vitamin B6^16^. Why such a low acid concentration is effective is a matter of speculation and requires further study. Current choline analyses, represented by *AOAC-Choline*, predominantly employ high acid concentrations (> 1.000 mol/L)^7,8,10,17^. Therefore, adequate hydrolysis of choline esters may be difficult to achieve. Moreover, *AOAC-Choline*^8^ faces safety issues owing to its high acid concentrations. Under the optimal hydrolysis conditions determined in this study, these issues are unlikely. In addition, the optimised conditions, in combination with sensitive and selective LC–MS/MS, are applicable to samples with low total choline content. For samples with lower choline content, matrix components coexisting with choline can affect the quantitative value of choline. Therefore, we validated the quantitative performance of the proposed method.

### Validation of total choline quantification performance

**Figure 4:**
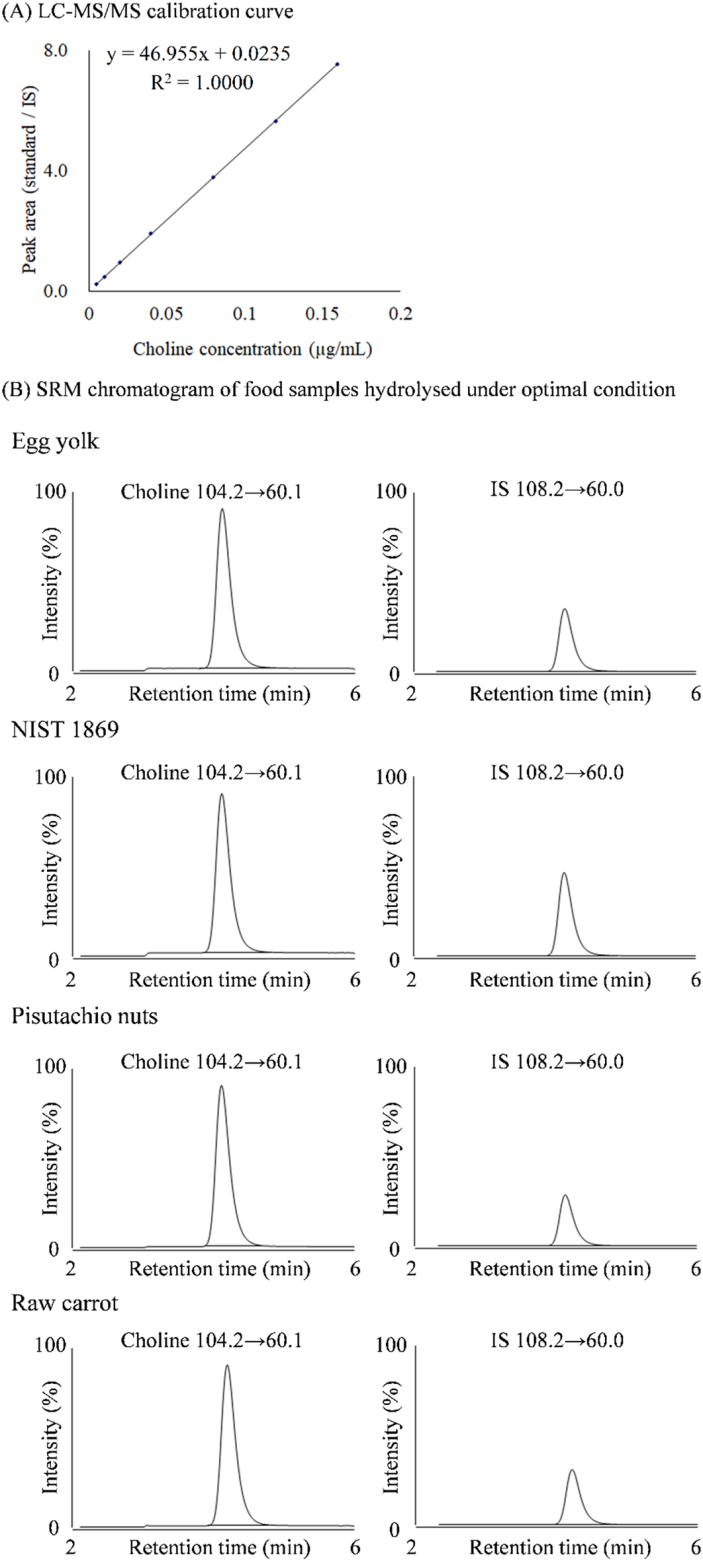
LC–MS/MS calibration curve and SRM chromatogram for food samples hydrolysed under optimal conditions. (**A**) The choline stock solution was diluted to 0.005, 0.010, 0.020, 0.040, 0.080, 0.120, and 0.160 μg/mL with 0.1% formic acid, after which IS was added at 0.040 μg/mL and analysed by LC–MS/MS. (**B**) The hydrolysed solution of each food sample (egg yolk, NIST 1869, pistachio nuts, and raw carrot) was injected into LC–MS/MS to analyses free choline. Detailed LC–MS/MS conditions are described in the Materials and methods. The intensity (%) in the graph for each food is based on choline SRM as 100%.

To validate the quantitative performance of the method combining optimal hydrolysis conditions and LC–MS/MS analysis, the accuracy and precision of the quantitation method of total choline in foods were evaluated (Supplementary Table S1 online) according to the AOAC guidelines^14^. With regard to food samples, because most previous studies have focused on the analysis of infant milk and whole-grain flour with high choline concentrations^9,18^, this study was conducted using a variety of foods with expectedly low and high choline contents that are commonly consumed in Japan. To detect choline (free form) sensitively and selectively, the LC–MS/MS analysis was performed using a PFP column for LC separation, with MS/MS detection in positive electrospray ionisation mode^13^. Under these conditions, the calibration curve for the free choline standard showed good linearity, with a coefficient of determination of 1.000 in the range 0.005‒0.160 µg/mL. The total choline in foods could be quantified without interference from other peaks of the food matrix components, as shown in the SRM chromatograms (Fig. 4).

The constructed method was used to hydrolyse 24 food samples, including the aforementioned egg yolk, raw chicken liver, soybean powder, and pistachio nuts, and their total choline content was determined (Table 1). Various quantitative parameters and values were obtained. The RSD_wr_ of all 24 samples ranged from 1.0 to 6.0%, and the HorRat_r_ values ranged from 0.3 to 1.4, which are within the acceptable range of the AOAC guidelines (0.5‒2.0). The average spike recovery rate of choline from food samples ranged from 90.4 to 110.4%, with most results within the acceptable range of the AOAC guidelines (85‒110%). The LOD and LOQ were 0.047 and 0.158 mg/100 g, respectively (for reference, the average total choline content in food is > 1 mg/100 g). These results clearly demonstrate that the present method is effective for determining the total choline content in a wide range of foods with low variability and high quantitation.

**Table 1:** Total choline content in 24 foods: average value, precision, and accuracy.

Sample	Total choline content (mean ± SD (*n* = 10), mg/100 g)	RSD_wr_ (%)	HorRat_r_	Average spike recovery rate (%)
Egg yolk	995.6 ± 9.8	1.0	0.5	106.2
Tarako (salted cod roe)	332.4 ± 9.6	2.9	1.2	108.4
Raw chicken liver	200.8 ± 4.3	2.1	0.8	99.9
Soybean powder	187.7 ± 3.5	1.8	0.7	105.7
Dried shiitake mushroom	184.2 ± 2.6	1.4	0.5	103.3
NIST 1869	163.2 ± 2.8	1.7	0.7	96.0
Raw fish salmon	147.9 ± 4.8	3.3	1.2	105.3
Pistachio nuts	123.2 ± 4.6	3.8	1.4	104.0
Dried seaweed	118.1 ± 3.0	2.6	0.9	105.9
Wheat flour	117.7 ± 2.3	2.0	0.7	109.8
Infant formula	89.3 ± 3.0	3.4	1.2	110.4
Fruit granola	71.6 ± 2.2	3.1	1.0	105.0
Raw pork fresh loin	59.2 ± 2.4	4.0	1.3	103.9
Yogurt	16.9 ± 0.8	4.8	1.3	108.0
Raw eggplant	13.1 ± 0.5	3.8	1.0	106.1
Raw spinach	11.2 ± 0.5	4.9	1.3	106.6
Infant liquid milk	10.9 ± 0.2	2.1	0.5	107.9
Adult nutritional ready-to-feed	10.7 ± 0.3	3.2	0.8	106.6
Raw carrot	9.8 ± 0.3	2.8	0.7	101.4
Raw tomato	8.6 ± 0.3	3.3	0.8	104.2
100% Orange juice	6.6 ± 0.3	4.0	0.9	102.1
Raw apple	6.5 ± 0.1	1.3	0.3	101.1
Honey	2.6 ± 0.1	2.0	0.4	98.2
Bottled green tea	0.3 ± 0.0	6.0	0.9	90.4

### Total choline content in foods and prospective applications

Egg yolk had the highest total choline content (995.6 mg/100 g) because it is rich in phosphatidylcholine. Cereal (wheat flour and soybean powder), seed (pistachio nuts), processed foods (fruit granola and infant formula), fish (raw fish salmon), and meats (fresh raw pork loin and raw chicken liver) had the next highest concentrations (50‒200 mg/100 g). The total choline concentrations in raw chicken liver, soybean powder, raw fish salmon, and pistachio nuts were 200.8, 187.7, 147.9, and 123.2 mg/100 g, respectively. This is because raw chicken liver, soybean powder, raw fish salmon, and pistachio nuts contain relatively high levels of phosphatidylcholine, phosphatidylcholine, glyceryl phosphorylcholine/sphingomyelin, and phosphocholine, respectively. Vegetables and fruits (raw apple, 100% orange juice, raw tomato, raw carrot, raw spinach, and raw eggplant) had relatively low total choline contents (≤ 20 mg/100 g). Among vegetables and fruits, eggplant showed a relatively high value, probably owing to the presence of acetylcholine^19^. Thus, the choline content in foods was consistent with the trends in previous reports^6^. Moreover, we found that foods unique to Japan (i.e., seaweed, dried shiitake mushroom, and tarako (cod roe)) are relatively rich in choline, suggesting that these foods may be a valuable source of choline.

Although the choline content of the foods was consistent with the trends in previous reports^6^, the quantitative values between our developed method and *AOAC-Choline* differ (Table 2). For the analysis of soybean powder, raw fish salmon, and pistachio nuts, which contain major choline esters and have relatively high total choline content, using both methods, the total choline content was found to be higher in all foods using our constructed method. This raises concerns about the insufficient hydrolysis of choline esters using *AOAC-Choline*, as described above. For NIST 1869, which consists mostly of free choline, the quantitative values obtained by the two methods were nearly identical, suggesting the validity of our hypothesis regarding hydrolysis. Additionally, the total choline contents measured by the developed method were higher than those in the database measured by *USDA-Choline* (Table 3). *USDA-Choline* measures four choline esters (phosphocholine, glyceryl phosphorylcholine, phosphatidylcholine, and sphingomyelin) individually and then sums them to calculate the total choline content. This may account for the lower values reported by *USDA-Choline* than those obtained by our constructed method, in which all types of choline esters were hydrolysed and measured. To further verify this, we need to actually measure and compare Japanese foods using *USDA-Choline* and the method we have constructed in this study. Furthermore, since our constructed method, *AOAC-Choline*, and *USDA-Choline* calculate the amount of choline per wet weight of the food, it would be desirable in the future to measure various foods not only by wet weight but also by dry weight and compare the measurements of the three methods.

**Table 2:** Total choline content in foods measured by our constructed method or *AOAC-Choline* ^8^.

Sample	Our constructed method (mean ± SD (*n* = 10), mg/100 g)	*AOAC-Choline* (mean ± SD (*n* = 3), mg/100 g)	*P*	Significance
Soybean powder	187.7 ± 3.5	171.3 ± 4.1	0.024	*
Raw fish salmon	147.9 ± 4.8	133.3 ± 4.3	0.015	*
Pistachio nuts	123.2 ± 4.6	106.6 ± 5.1	0.038	*
NIST 1869 (Total choline content as measured by *AOAC-Choline* attached to this product: 161.2 ± 6.4 mg/100 g)	163.2 ± 2.8	160.7 ± 3.8	0.421	–

*Indicates significant difference (*P* < 0.05)—indicates not significant difference.

**Table 3:** Comparison of total choline content in foods measured by our constructed method and values listed in the database measured by *USDA-Choline* ^6^.

Sample	Our constructed method (mean ± SD (*n* = 10), mg/100 g)	USDA nutrient database number	*USDA-Choline* (mg/100 g)
Egg yolk	995.6 ± 9.8	01125	680.0
Raw chicken liver	200.8 ± 4.3	05027	190.0
Soybean powder	187.7 ± 3.5	16108	120.0
Raw fish salmon	147.9 ± 4.8	15236	79.0
Pistachio nuts	123.2 ± 4.6	12652	71.0
Raw pork fresh loin	59.2 ± 2.4	10066	60.0
Yogurt	16.9 ± 0.8	01117	15.0
Raw carrot	9.8 ± 0.3	11124	8.8
Raw tomato	8.6 ± 0.3	11529	6.7
100% Orange juice	6.6 ± 0.3	09202	8.4
Raw apple	6.5 ± 0.1	09003	3.4
Honey	2.6 ± 0.1	19296	2.2

**Figure 5:**
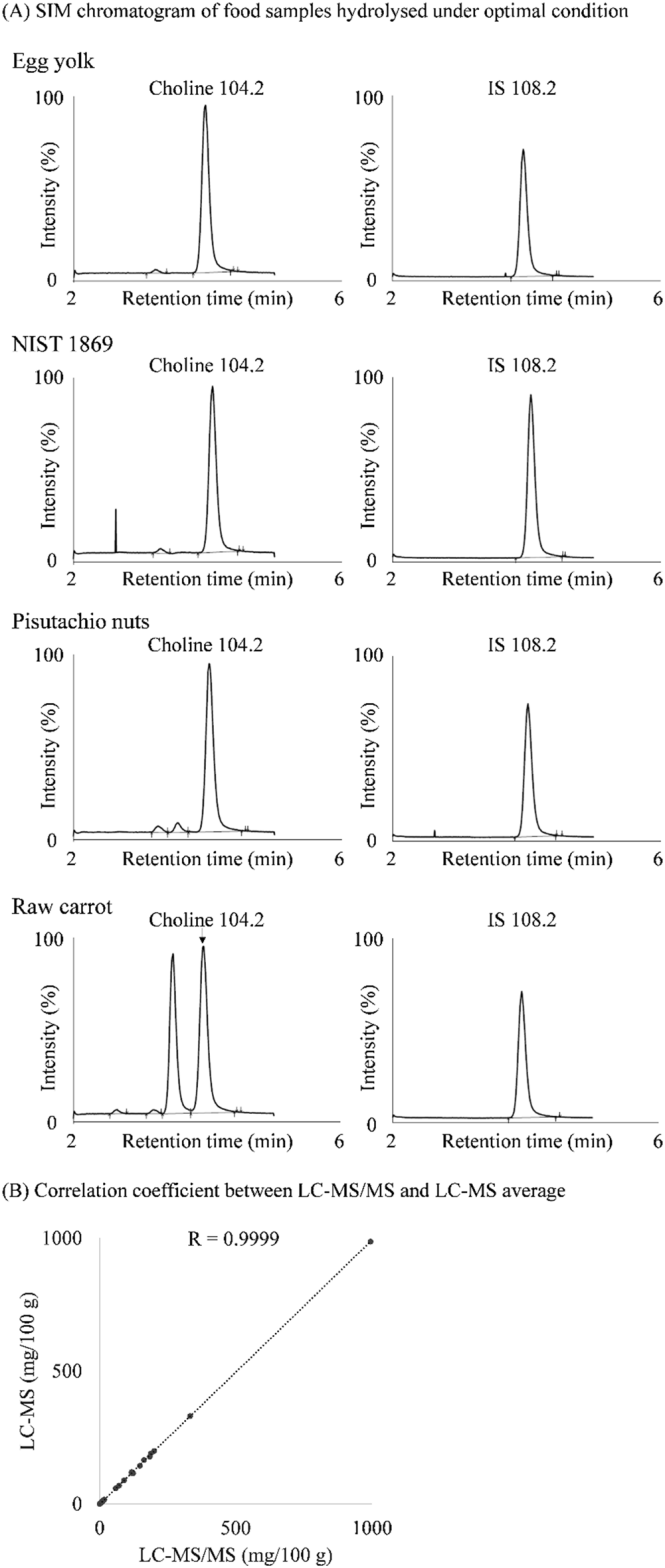
SIM chromatogram of food samples hydrolysed under optimal conditions and correlation coefficient for average values from LC–MS/MS and LC–MS. (**A**) The hydrolysed solution of each food sample (egg yolk, NIST 1869, pistachio nuts, and raw carrot) was injected into LC–MS to analyse free choline. Detailed LC–MS conditions are described in the Materials and methods. The intensity (%) in the graph for each food is based on choline SIM as 100%. (**B**) The 24 foods were measured 10 times using both LC–MS/MS and LC–MS, and the average value was calculated. The average value for yogurt determined using LC–MS was calculated from six analyses. The average value from LC–MS/MS is plotted on the Y axis of the graph, and the average value from LC–MS is plotted on the X axis of the graph; the correlation coefficient was determined.

On a side note, using LC–MS instead of LC–MS/MS, we were able to calculate the total choline content in most foods. Although the sensitivity and selectivity of LC–MS were somewhat less, the correlation between the LC–MS/MS and LC–MS values for the 24 foods showed good agreement (Fig. 5).

## Conclusions

Overall, an analytical method for the accurate determination of the total choline in various foods was constructed by optimising the acid hydrolysis conditions and using sensitive and selective LC–MS/MS. Because LC–MS can also be used instead of LC–MS/MS, measurements can be performed in many laboratories. As mentioned above, current methods represented by *AOAC-Choline* may underestimate the total choline in various foods. Therefore, we recommend re-evaluation of the literature data using our constructed method. This reassessment is expected to allow for more reliable evaluation of choline intake, which in turn will contribute to health maintenance.

### Supplementary Information

Supplementary Information.

## Data Availability

The datasets used and/or analysed in the current study are available from the corresponding author upon reasonable request.
