# Supplementary material for: Optimisation of acid hydrolysis conditions of choline esters and mass spectrometric determination of total choline in various foods
Source: Sci Rep. 2024 Aug 2;14:17960. doi: 10.1038/s41598-024-69008-z (PMC11297186; doi:10.1038/s41598-024-69008-z)
Supplement: Supplementary file 1 — Supplementary Information. [file 41598_2024_69008_MOESM1_ESM.pdf]

Optimisation of acid hydrolysis conditions of choline esters and mass spectrometric determination of total choline in various foods

Author information

Yoshinari Hirakawa[1,2 *], Kazuhiro Fujita[1], Masako Katayama[1], Toshiaki Yokozeki[1], Yushi Takahashi[1], Izumi Yoshida[1], Kiyotaka Nakagawa[2].

1 Japan Food Research Laboratories, 7-4-41 Saitoasagi, Ibaraki, Osaka 567-0085, Japan

2 Laboratory of Food Function Analysis, Graduate School of Agricultural Science, Tohoku University, 468-1 Aramaki Aza Aoba, Aoba-ku, Sendai, 980–8572, Japan

[*]Corresponding author**:** Japan Food Research Laboratories, 7-4-41 Saitoasagi, Ibaraki, Osaka 567-0085, Japan; Tel.: +81 72 641 8958; Fax: +81 72 641 8969
E-mail address: hirakaway@jfrl.or.jp

Supplementary Table S1. Parameters for obtaining valid data according to AOAC guidelines[14]

Validation parameter	Sample	Measurement	Evaluation
Linearity	Standard solution (0.005–0.160 µg/mL)	-	Coefficient of determination
Precision	24 food samples	n = 10	RSD_{wr} and $HorRat_r$ values
Accuracy	24 food samples	n = 3	Average spike recovery rate
LOD and LOQ	Bottled green tea	n = 10	LOD = 3 × SD, LOQ = 10 × SD

Condition 1
(*AOAC-Choline*[8])

・Sample
A 1 mL (*W*) aliquot of the test solution (n=3)
Phosphocholine (50.00 mg/100 mL),
glyceryl phosphorylcholine (90.60 mg/100 mL),
phosphatidylcholine (1985 mg/100 mL), and
sphingomyelin (356.2 mg/100 mL)

・Acid addition
Add 25 mL of 3.650 mol/L HNO_3 aqueous solution

・Acid hydrolysis
120 °C microwave (40 min, 1000 W)

・NaOH addition
Neutralise to pH 6.8

・Dilution
Make the solution up to 100 mL (V_a) with water
Filter through a filter paper
Dilute with water; dilution ratio ***D*** (1–200)

・IS addition
Transfer 1 mL (V_b) of the diluted solution to a flask
Add 0.25 mL of IS (1.000 µg/mL water)
Make up to 5 mL (V_c) with water

・Solid-phase column (InertSep WCX-FF)
Load 4 mL (V_d) of the solution onto the column

・Washing
Wash the column with 4 mL of water

・Elution
Elute choline with 4 mL of 0.1% formic acid aqueous solution
Adjust the final volume to 5 mL (V_e) with 0.1% formic acid

・Analysis
Inject 1 µL into the LC-MS/MS
Calculate the choline concentration ***C*** (µg/mL)

$$\text{Test solution Choline (mg/100 mL)} = \frac{C}{W} \times \frac{V_a}{V_b} \times \frac{V_c}{V_d} \times V_e \times D \times 0.1$$

0.1 (conversion factor from µg/mL to mg/100 mL)

Condition 2
(*AOAC-Niacin* (nicotinic acid and nicotinamide)[11])

・Sample
A 1 mL (*W*) aliquot of the test solution (n=3)
Phosphocholine (50.00 mg/100 mL),
glyceryl phosphorylcholine (90.60 mg/100 mL),
phosphatidylcholine (1985 mg/100 mL), and
sphingomyelin (356.2 mg/100 mL)

・Acid addition
Add 50 mL of 0.500 mol/L H_2SO_4 aqueous solution

・Acid hydrolysis
121 °C autoclave (30 min)

・NaOH addition
Neutralise to pH 6.8

・Dilution
Make the solution up to 100 mL (V_a) with water
Filter through a filter paper
Dilute with water; dilution ratio ***D*** (1–200)

・IS addition
Transfer 1 mL (V_b) of the diluted solution to a flask
Add 0.25 mL of IS (1.000 µg/mL water)
Make up to 5 mL (V_c) with water

・Solid-phase column (InertSep WCX-FF)
Load 4 mL (V_d) of the solution onto the column

・Washing
Wash the column with 4 mL of water

・Elution
Elute choline with 4 mL of 0.1% formic acid aqueous solution
Adjust the final volume to 5 mL (V_e) with 0.1% formic acid

・Analysis
Inject 1 µL into the LC-MS/MS
Calculate the choline concentration ***C*** (µg/mL)

$$\text{Test solution Choline (mg/100 mL)} = \frac{C}{W} \times \frac{V_a}{V_b} \times \frac{V_c}{V_d} \times V_e \times D \times 0.1$$

0.1 (conversion factor from µg/mL to mg/100 mL)

Condition 3
(*AOAC-B6* (pyridoxine, pyridoxal and pyridoxamine)[12])

・Sample
A 1 mL (*W*) aliquot of the test solution (n=3)
Phosphocholine (50.00 mg/100 mL),
glyceryl phosphorylcholine (90.60 mg/100 mL),
phosphatidylcholine (1985 mg/100 mL), and
sphingomyelin (356.2 mg/100 mL)

・Acid addition
Add 70 mL of 0.055 mol/L HCl aqueous solution

・Acid hydrolysis
121 °C autoclave (4 h)

・Dilution*
Make the solution up to 100 mL (V_a) with water
Filter through a filter paper
Dilute with 0.1% formic acid; dilution ratio ***D*** (5–1000)

・IS addition
Transfer 0.9 mL (V_b) of the diluted solution to a flask
Add 0.1 mL of the IS (0.400 µg/mL, 0.1% formic acid) to 1 mL of the solution

・Analysis
Inject 1 µL into the LC-MS/MS
Calculate the choline concentration ***C*** (µg/mL)

$$\text{Test solution Choline (mg/100 mL)} = \frac{C}{W} \times \frac{V_a}{V_b} \times V_c \times D \times 0.1$$

$$\text{Food samples Choline (mg/100 g)} = \frac{C}{W} \times \frac{V_a}{V_b} \times V_c \times D \times 0.1$$

0.1 (conversion factor from µg/mL to mg/100 mL or mg/100 g)

Optimisation of hydrolysis based on Condition 3

・Sample
A 1 mL (*W*) aliquot of the test solution (n=3)
Phosphocholine (50.00 mg/100 mL),
glyceryl phosphorylcholine (90.60 mg/100 mL),
phosphatidylcholine (1985 mg/100 mL), and
sphingomyelin (356.2 mg/100 mL)

・Acid addition
Add 70 mL of 0.010, **0.055**, and 0.100 mol/L HCl aqueous solution

・Acid hydrolysis
121 °C Autoclave (0.5, 4, **8**, and 16 h)

・Dilution*
The method after dilution is the same as described in Condition 3

Optimisation of hydrolysis based on Condition 3

・Sample
A 0.5 g (*W*) crushed food sample (n=3)
Egg yolk, raw chicken liver, soybean powder, and pistachio nuts

・Acid addition
Add 70 mL of **0.055** mol/L HCl aqueous solution

・Acid hydrolysis
121 °C Autoclave (4, **8**, and 16 h)

・Dilution*
The method after dilution is the same as described in Condition 3

Optimal hydrolysis condition

・Sample
A crushed 24 food sample (n=10)
0.5 g (*W*); wheat flour, soybean powder, pistachio nuts, dried shiitake mushroom, dried seaweed, raw fish salmon, tarako, raw pork fresh loin, raw chicken liver, adult nutritional ready-to-feed, infant liquid milk, infant formula, yogurt, fruit granola, egg yolk, and NIST 1869
1.0 g (*W*); raw tomato, raw carrot, raw spinach, raw eggplant, raw apple, 100% orange juice, bottled green tea, and honey

・Acid addition
Add 70 mL of **0.055** mol/L HCl aqueous solution

・Acid hydrolysis
121 °C Autoclave (**8** h)

・Dilution*
The method after dilution is the same as described in Condition 3

Supplementary Fig. S1 Detailed flow chart of acid hydrolysis studies. In addition to the studies, some foods (soybean powder, pistachio nuts, raw fish salmon, and NIST 1869; 0.5 g) were hydrolysed under *AOAC-Choline*[8] (Condition 1) to measure choline (n=3) and compared to choline determined by the method constructed in this study. Also, 24 food samples were hydrolysed under the optimal condition to measure choline (n=10) using LC-MS.
